# Supplementary material for: Multi-omics of barley Fusarium Head Blight converge on pathogen-triggered biosynthesis of aromatic amino acid derived chemical defense compounds
Source: Stress Biol. 2026 Jun 1;6(1):41. doi: 10.1007/s44154-026-00313-5 (PMC13226754; doi:10.1007/s44154-026-00313-5)
Supplement: Supplementary file 1 — Supplementary Material 1. [file 44154_2026_313_MOESM1_ESM.docx]

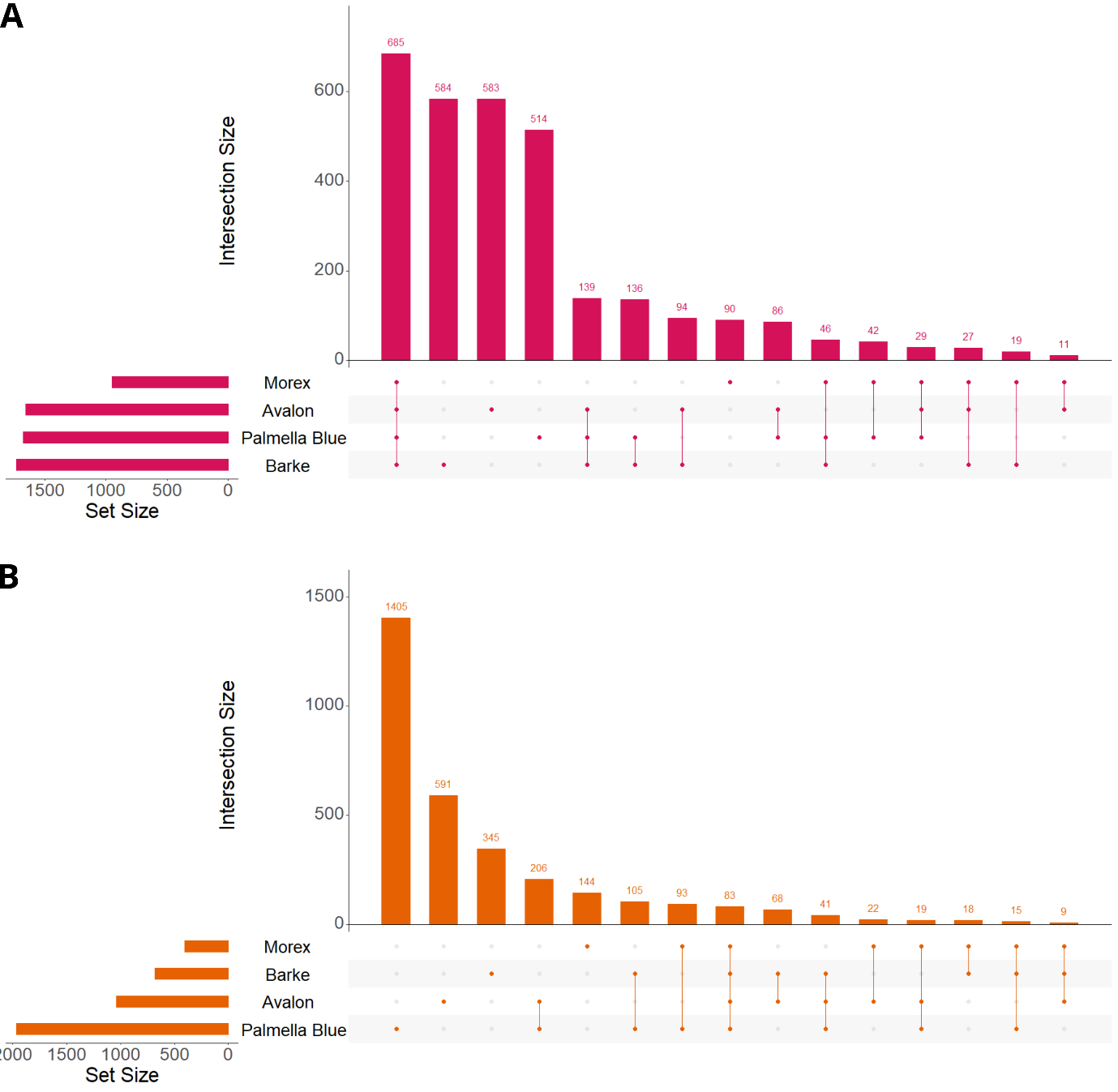


**Fig. S1**: UpSet plot illustrating the intersections among the DEGs (**A**) and DEPs (**B**) identified in four barley cultivars Avalon, Barke, Morex and Palmella Blue. Horizontal bars on the left represent the total number of DEPs or DEGs in each individual cultivar. The matrix indicates which sets participate in each intersection, with connected dots marking a given combination. Vertical bars above the matrix show the size of each intersection.


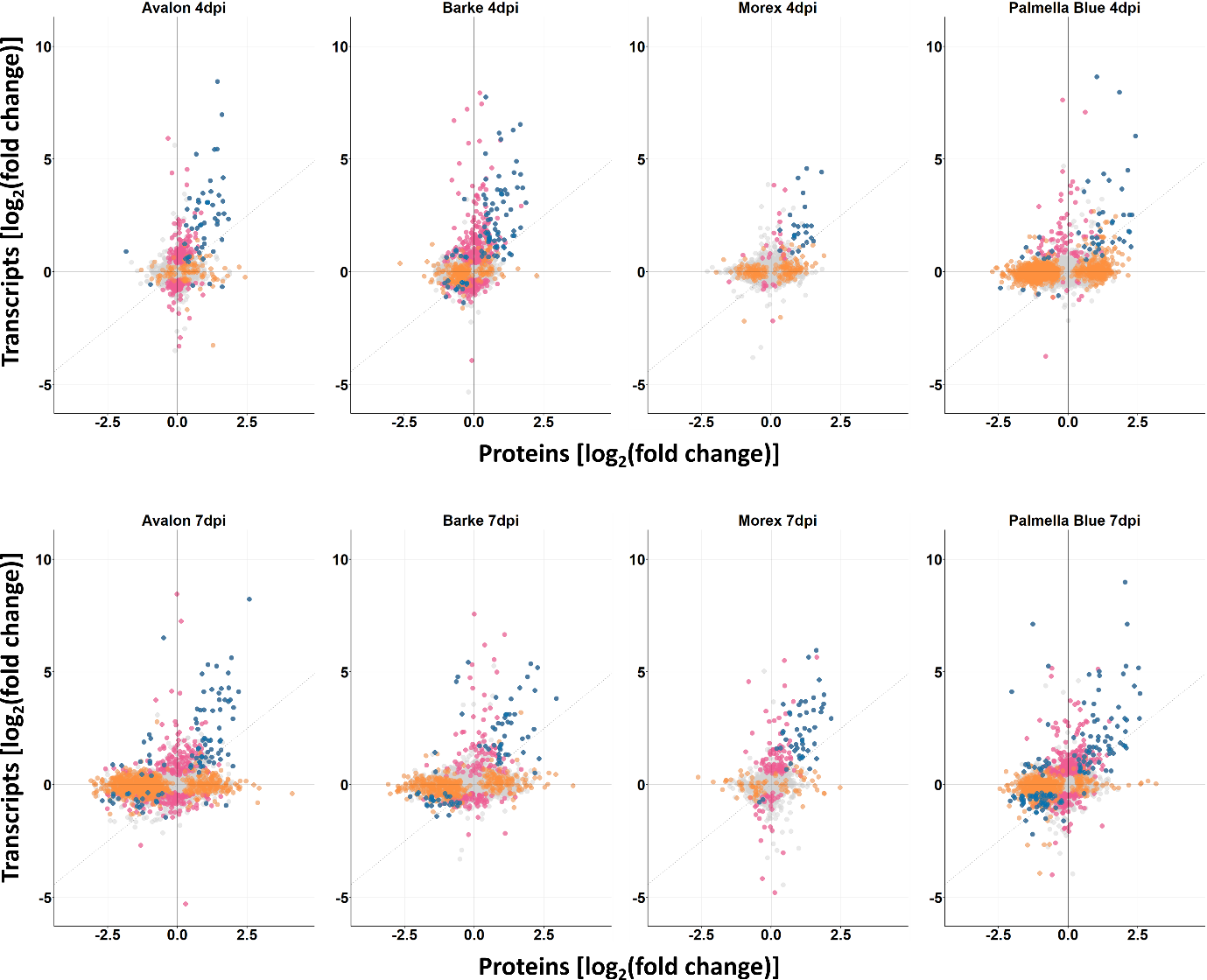


**Fig. S2** Comparison of fold changes of transcripts and corresponding proteins in barley cultivars Avalon, Barke, Morex, and Palmella Blue at 4 and 7 days post inoculation. Pink: Differentially expressed genes (DEGs). Orange: Differentially abundant proteins (DEPs). Blue: DEP/DEG pairs. Inoculation was performed around mid-anthesis. Infected samples were compared with mock-treated controls of the same cultivar and time-point. For each cultivar, treatment, and time point, four replicates were collected, each consisting of three pooled spikes.


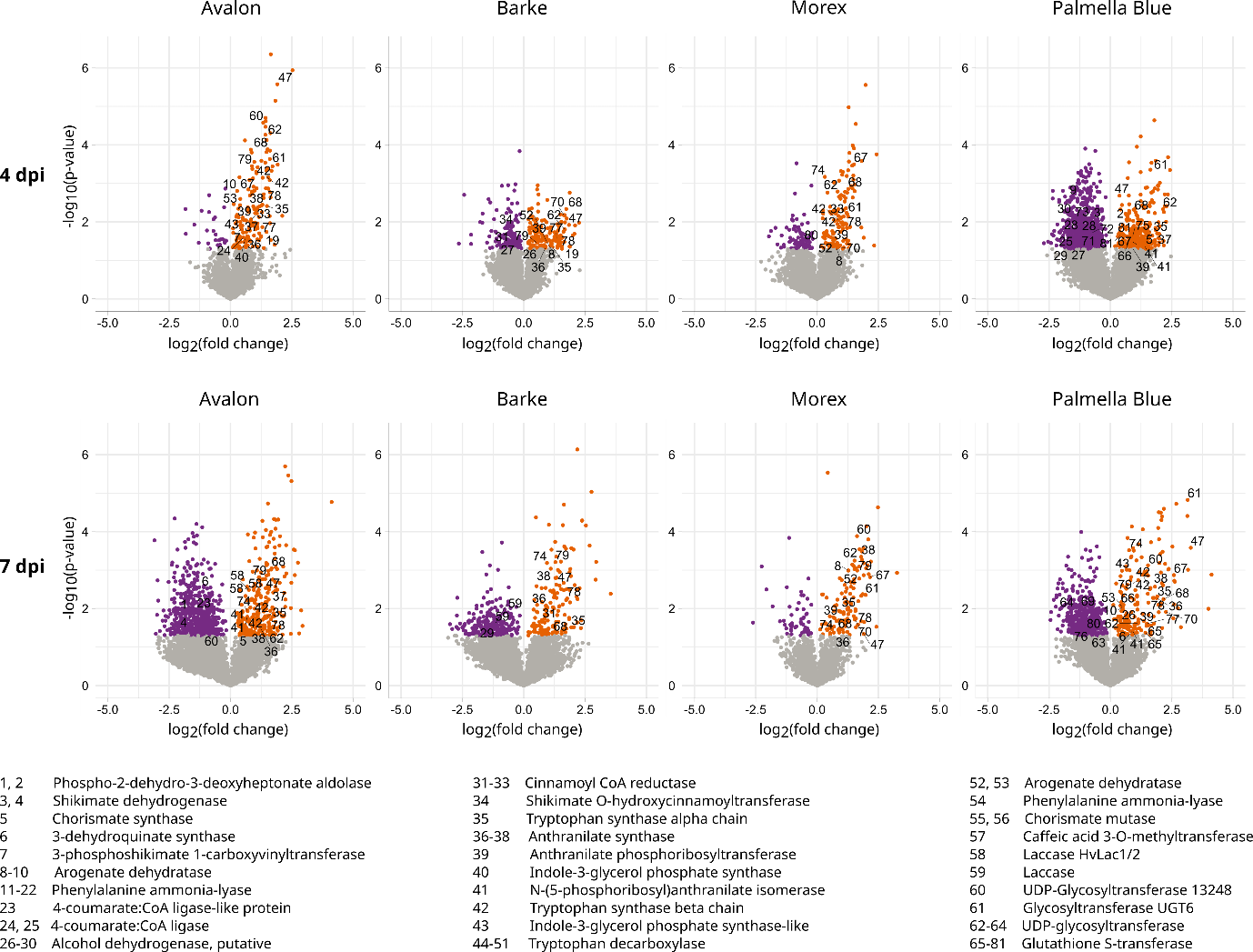


**Fig. S3** Volcano plots of the differential abundance of proteins in barley cultivars Avalon, Barke, Morex, and Palmella Blue at four and seven days post inoculation with *F. culmorum* spore solution in comparison with mock-treated controls. Inoculation was performed around mid-anthesis. Infected samples were compared with mock-treated controls of the same cultivar and time-point. For each cultivar, treatment, and time point, four replicates were collected, each consisting of three pooled spikes. Volcano plots show the log_2_(fold change) values in relation to the negative log_10_(p-value).


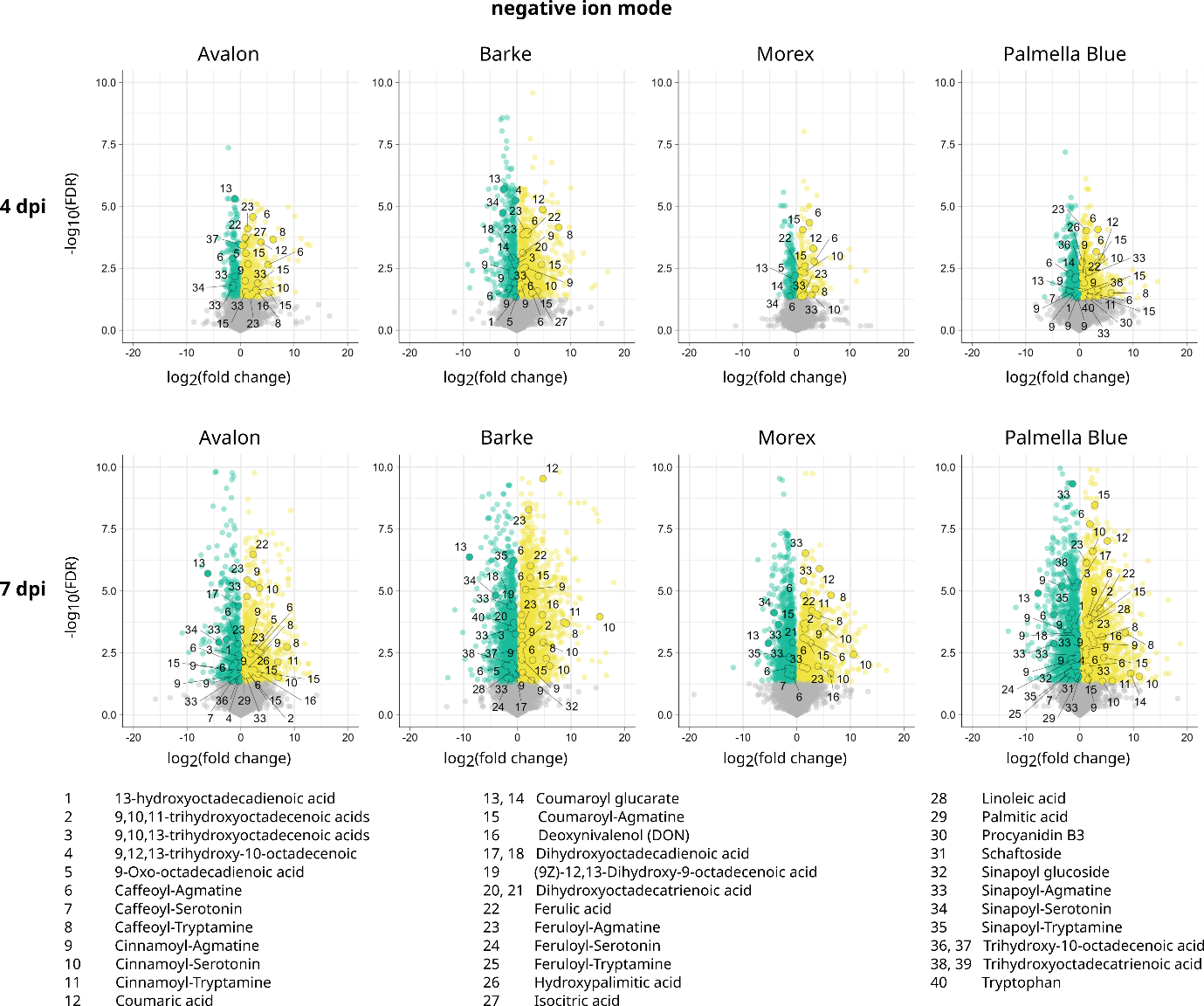


**Fig. S4** Volcano plots of the differential abundance of metabolic features in barley cultivars Avalon, Barke, Morex, and Palmella Blue at four and seven days post inoculation with *F. culmorum* spore solution in comparison with mock-treated controls. Metabolic features were measured via mass spectrometry in negative ion mode. Inoculation was performed around mid-anthesis. Infected samples were compared with mock-treated controls of the same cultivar and time-point. For each cultivar, treatment, and time point, four replicates were collected, each consisting of three pooled spikes. Volcano plots show the log_2_(fold change) values in relation to the negative log_10_(FDR).


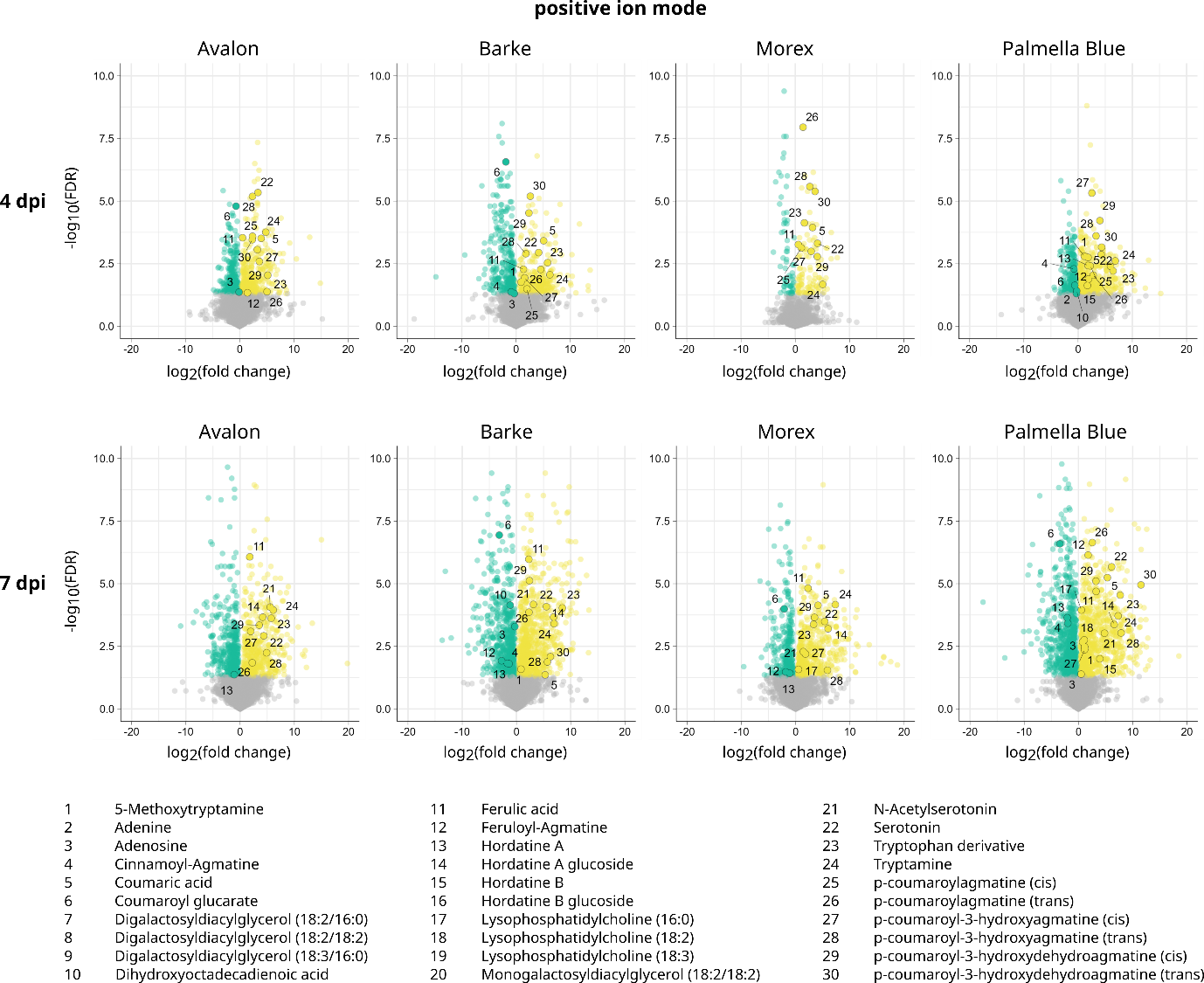


**Fig. S5** Volcano plots of the differential abundance of metabolic features in barley cultivars Avalon, Barke, Morex, and Palmella Blue at four and seven days post inoculation with *F. culmorum* spore solution in comparison with mock-treated controls. Metabolic features were measured via mass spectrometry in positive ion mode. Inoculation was performed around mid-anthesis. Infected samples were compared with mock-treated controls of the same cultivar and time-point. For each cultivar, treatment, and time point, four replicates were collected, each consisting of three pooled spikes. Volcano plots show the log_2_(fold change) values in relation to the negative log_10_(FDR).


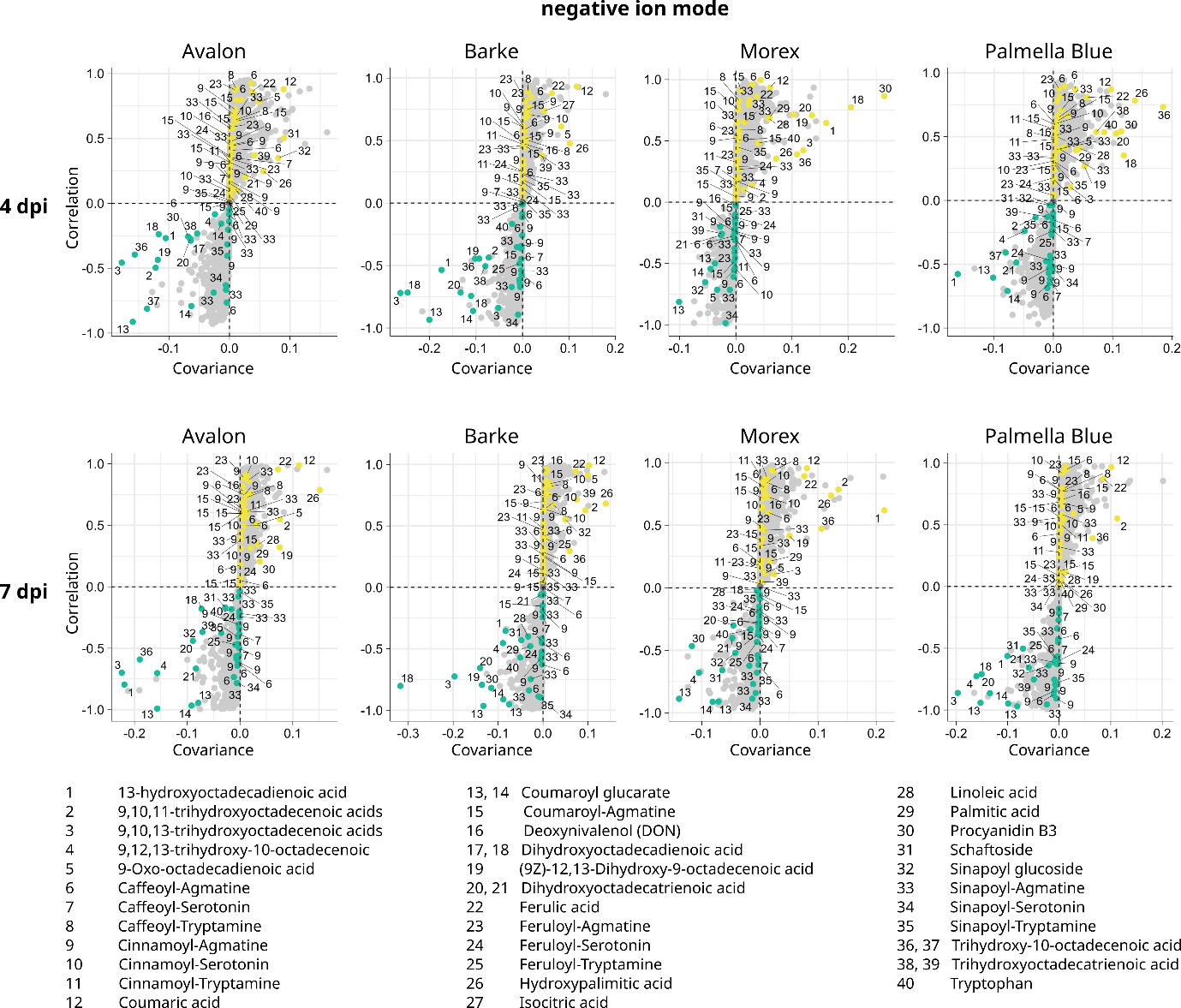


**Fig. S6** S-plots of the differential abundance of metabolic features in barley cultivars Avalon, Barke, Morex, and Palmella Blue at four and seven days post inoculation with *F. culmorum* spore solution in comparison with mock-treated controls. Metabolic features were measured via mass spectrometry in negative ion mode. Inoculation was performed around mid-anthesis. Infected samples were compared with mock-treated controls of the same cultivar and time-point. For each cultivar, treatment, and time point, four replicates were collected, each consisting of three pooled spikes. S‑plots show group differences as calculated via orthogonal partial least squares discriminant analysis (OPLS-DA).


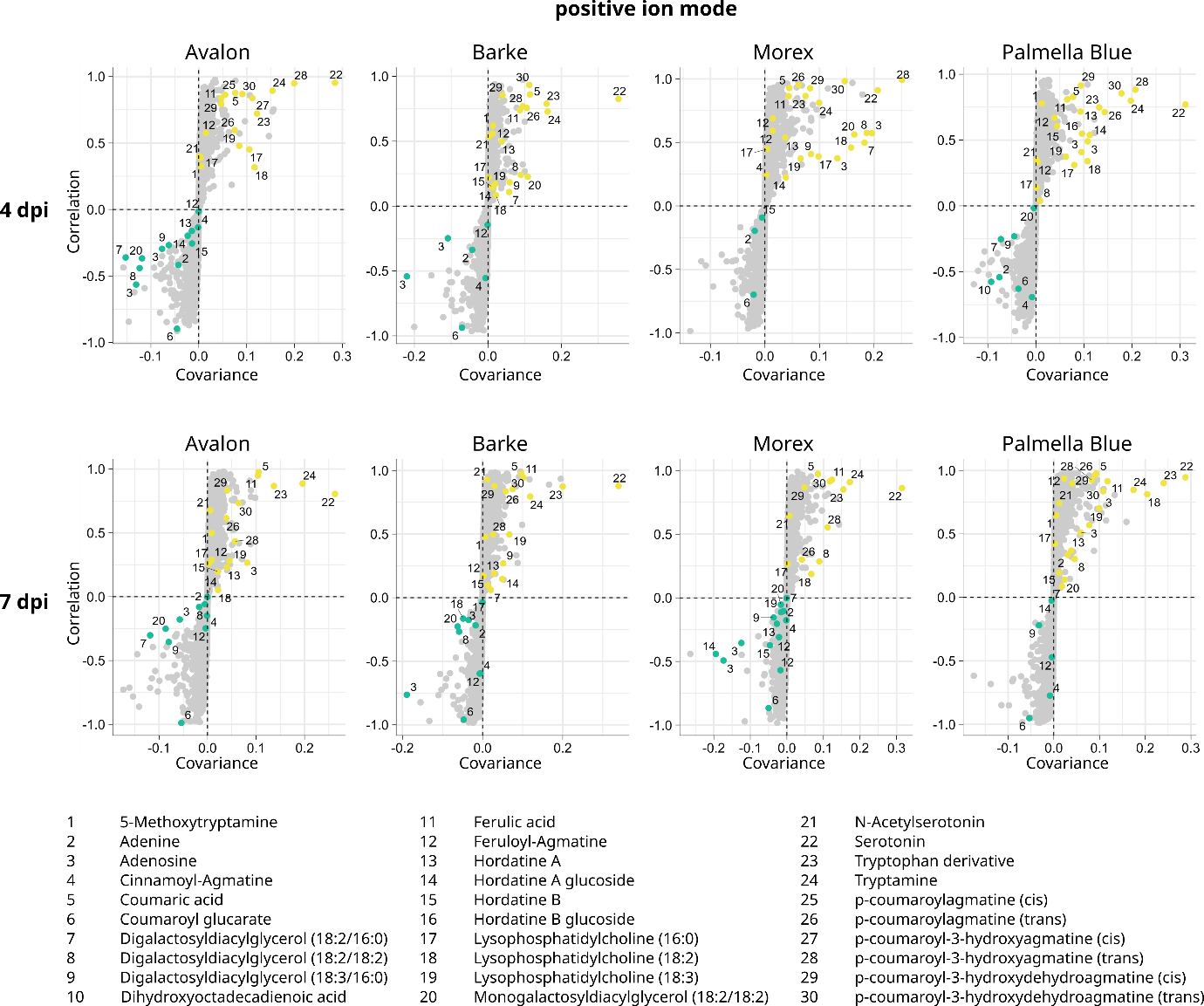


**Fig. S7** S-plots of the differential abundance of metabolic features in barley cultivars Avalon, Barke, Morex, and Palmella Blue at four and seven days post inoculation with *F. culmorum* spore solution in comparison with mock-treated controls. Metabolic features were measured via mass spectrometry in positive ion mode. Inoculation was performed around mid-anthesis. Infected samples were compared with mock-treated controls of the same cultivar and time-point. For each cultivar, treatment, and time point, four replicates were collected, each consisting of three pooled spikes. S‑plots show group differences as calculated via orthogonal partial least squares discriminant analysis (OPLS-DA).
